# Genomic Selection for Late Blight and Common Scab Resistance in Tetraploid Potato (*Solanum tuberosum*)

**DOI:** 10.1534/g3.118.200273

**Published:** 2018-05-24

**Authors:** Felix Enciso-Rodriguez, David Douches, Marco Lopez-Cruz, Joseph Coombs, Gustavo de los Campos

**Affiliations:** * Department of Plant, Soil, and Microbial Sciences; † Department of Epidemiology & Biostatistics; ‡ Department of Statistics & Probability; § Institute for Quantitative Health Science and Engineering, Michigan State University, East Lansing, Michigan, 48824

**Keywords:** Potato, Late blight, Common scab, Disease resistance, Genome-Wide Regression, Genomic Selection, Polyploidy, Bayesian, BGLR

## Abstract

Potato (*Solanum tuberosum*) is a staple food crop and is considered one of the main sources of carbohydrates worldwide. Late blight (*Phytophthora infestans*) and common scab (*Streptomyces scabies*) are two of the primary production constraints faced by potato farming. Previous studies have identified a few resistance genes for both late blight and common scab; however, these genes explain only a limited fraction of the heritability of these diseases. Genomic selection has been demonstrated to be an effective methodology for breeding value prediction in many major crops (*e.g.*, maize and wheat). However, the technology has received little attention in potato breeding. We present the first genomic selection study involving late blight and common scab in tetraploid potato. Our data involves 4,110 (Single Nucleotide Polymorphisms, SNPs) and phenotypic field evaluations for late blight (n=1,763) and common scab (n=3,885) collected in seven and nine years, respectively. We report moderately high genomic heritability estimates (0.46 ± 0.04 and 0.45 ± 0.017, for late blight and common scab, respectively). The extent of genotype-by-year interaction was high for late blight and low for common scab. Our assessment of prediction accuracy demonstrates the applicability of genomic prediction for tetraploid potato breeding. For both traits, we found that more than 90% of the genetic variance could be captured with an additive model. For common scab, the highest prediction accuracy was achieved using an additive model. For late blight, small but statistically significant gains in prediction accuracy were achieved using a model that accounted for both additive and dominance effects. Using whole-genome regression models we identified SNPs located in previously reported hotspots regions for late blight, on genes associated with systemic disease resistance responses, and a new locus located in a WRKY transcription factor for common scab.

The potato (*Solanum tuberosum* L.) is considered the sixth most important agricultural commodity worldwide after sugar cane, maize, rice, wheat and milk. In 2014, the global production of potatoes exceeded 385 million tons, positioning China as the largest producer with more than 66 million tons, followed by Russia, India and the United States (FAOSTAT 2016). As a staple food, this crop represents one of the main sources of carbohydrates, fiber, minerals and vitamins, providing essential nutrients and energy needed for healthy body development and function (Kolasa 1993; Drewnowski and Rehm 2013).

Despite its great economic and food security importance, potatoes face high production losses caused mainly by biotic factors. Among them, pathogens such as late blight (*Phytophthora infestans* (Mont.) de Bary), represent the most devastating disease for potato worldwide. Late blight infects vegetative tissues, typically killing the entire plant, within 7 to 10 days. This pathogen accounts for annual losses of 16% of the global potato production (Haverkort *et al.*, 2009). Under increasingly variable weather conditions, late blight incidence is expected to escalate worldwide, affecting mainly highlands in developing countries (Sparks *et al.* 2014).

Soil-borne pathogens such as common scab (*Streptomyces scabies* Thaxter), reduces the potato quality and marketability by causing superficial lesions on the tuber surface (Dees and Wanner 2012). Susceptibility to common scab is dependent upon genotype, time and environmental conditions (Wanner 2006; Wanner and Kirk 2015), having a negative impact mainly in underground tissues in development, such as stolons and tubers. This pathogen has spread worldwide and due to its saprophyte nature, (common scab can survive in winter), thus becoming a permanent source of inoculum for the next planting seasons, causing losses up to $100/Ha (Wanner and Kirk 2015).

Pathogen infection can be controlled by using protectants or systematic fungicides; however, there methods can be ineffective if the environmental conditions favor pathogen dispersion (Nowicki *et al.* 2011) or the emergence of fungicide-resistant genotypes (Pomerantz *et al.* 2014). The most effective way to control the incidence of late blight and common scab in potatoes is through the generation of resistant varieties (Ahn and Park 2013). However, breeding for resistant varieties via phenotypic selection can take up to 15 years, making traditional breeding time-consuming and sometimes ineffective against fast-evolving pathogens (The Potato Genome Sequencing Consortium 2011; Lozano *et al.* 2012).

Marker-assisted (Barone 2004) and genomic selection (GS) strategies (Meuwissen *et al.* 2001) can accelerate the process of breeding disease resistance. Several studies on late blight and common scab resistance have reported variants conferring resistance to these pathogens; however, most of the genomic research has focused on late blight (Gebhardt *et al.* 2004; Malosetti *et al.* 2007; Muktar *et al.* 2015; Mosquera *et al.* 2016; Braun *et al.* 2017a) and are largely based on phenotype-single marker association analyses. To the best of our knowledge, no study so far has considered the use of GS for breeding resistance to late blight and common scab in potato. Therefore, in this article, we use Whole-Genome Regression methods commonly used in GS to: (i) study important features of the genetic architecture of resistance to late blight and common scab (including trait heritability, extent of genetic-by-environment interactions (G×E) and the importance of non-additive effects), (ii) identify large-effect variants contributing to resistance to late blight and scab, and (iii) assess the prediction accuracy of GS for resistance to those two pathogens.

Our data involves (up to) nine years of field evaluations for late blight and common scab at two Michigan State University’s (MSU) research centers. We considered models that accounted for additive effects and various forms of dominance and evaluated two different statistical methods. Our results suggest that sizable fraction of the inter-individual differences in disease resistance (∼46% for late blight and 45% for common scab) can be captured by the SNP set used in the study. The extent of G×E was low for common scab and high for late blight. We found that additive models can capture more than 90% of the genetic variance. We report large-effect SNPs contributing to late blight resistance in chromosomes V and IX, that have been previously reported to harbor resistance genes to this pathogen. We also report the first SNP associated with common scab resistance, located on chromosome IX, and positioned in a transcription factor known for its role in systemic defense and resistance responses. Our results demonstrate that genomic selection can yield moderately accurate prediction of disease resistance for genotypes that have been not evaluated in field trials. Thus, GS could be used for rapid cycling selection for resistance to both late blight and common scab in tetraploid potato.

## Materials and Methods

### Data

Data were collected from early generation and advanced tetraploid potato genotypes derived from bi-parental crosses at the MSU potato breeding program. Additional advanced breeding genotypes from other United States breeding programs and reference varieties were also included. The available genotypes (n = 381) represent different market classes for fresh market, chip-processing, and russet-type fresh market and processing varieties. These genotypes were evaluated in field trials that included annual selections from MSU’s potato breeding program, where each year poorly performing genotypes were replaced with new genotypes, while maintaining control genotypes during consecutive years.

***Late blight field resistance trials*** (273 genotypes and a total of 1,763 disease records) were conducted in inoculated foliar field trials during seven years (2010-2015 and 2017) at the MSU’s Clarksville Research Center (Clarksville, MI). Potato seed tubers were hand planted early- to mid-June as four-plant hills in 1.5 m plots in a randomized complete block design with one to three replicates. Late blight-susceptible rows and plots were planted around the perimeter and between blocks to promote an even late blight distribution in the field. After approximately 60 days, all plots were inoculated with a zoospore suspension of late blight at 3x10^6^ spores/mL at the end of July or beginning of August of each year. Over the 7-year period, different isolates were used to infect the trial depending on the prominent isolate in the region. The *P. infestans* strain (clonal lineage) detected in each year in the trial can be found in Table S1 in File S4. Following inoculation, plots were rated visually for the percentage of foliar area affected by late blight. Ratings were taken at 3 to 7-day intervals, based on the rate of disease progression during 35-50 days post inoculation (DPI). Finally, the percent defoliation data were used to calculate the relative area under the disease progress curve - RAUDPC (Fry 1978).

***Common scabfield resistance trials*** (370 genotypes and a total of 3,885 disease records) were conducted under field conditions during nine years (2009-2017) in a disease nursery at the MSU’s Montcalm Research Center (Lakeview, MI). The field was inoculated with common scab from aggressive Michigan isolates, and has been cultivated for high disease pressure for the past nine years. The trials were planted in a randomized complete block design consisting of one to four replications of five-hill plots. After harvesting, mature tubers in plots were assessed for their overall plot disease rating scale of 0-5. The rating was based on a combined score for common scab coverage and lesion severity in which a rating of 0 indicates zero infection and 3.0 or greater scores represent highly susceptible genotypes with >50% infection and severe pitted lesions (Driscoll *et al.* 2009).

***SNP genotypes*** were obtained using the Infinium 8303 Potato Array. Plant DNA was isolated from young potato leaves or tubers using the Qiagen DNeasy Plant Mini Kit (Qiagen, Germany), following manufacturer’s instructions. DNA was quantified using the Quant-iT PicoGreen dsDNA Assay kit (Invitrogen, San Diego, CA). Genotype scoring was performed using the GenomeStudio software (Illumina, San Diego, CA). The tetraploid SNP calling was performed as per Hirsch *et al.* (2013), using a custom tetraploid genotype calling based on theta values from the Illumina GenomeStudio (Illumina, San Diego, CA) and subsequently filtered, removing poor quality markers. SNPs were coded by counting the number of copies of a reference allele (*e.g.*, B) where 0 denotes fully homozygous allele (AAAA), 1-3 represent heterozygous genotypes (AAAB, AABB, ABBB, respectively) and 4 the other homozygous genotype (BBBB). The genotype file was filtered by retaining SNPs with minor allele frequency (MAF) >0.05 and missing rate <0.15. The remaining missing SNP-based genotypes were imputed with the SNP means. The final number of SNPs that passed the quality filtering were 4,110.

We compared the observed and expected rates of heterozygous loci, the later derived under the assumption of Hardy-Weinberg (HW) equilibrium. Averaged across loci, the observed rates of heterozygosity (0.663) was only slightly higher than the one predicted from estimated allele frequencies (0.647). The regression of the observed and expected frequency of heterozygous loci had an estimated slope of 0.98 (SE = 0.0025) and a R^2^ of 0.974. Moreover, we did not identify any clear outlier SNP that may have indicated a significant deviation of the observed frequency of heterozygous relative to the one predicted from the estimated allele frequency of the locus.

***Genomic relationships*** were computed from centered and scaled SNP-based genotypes according to VanRaden (2008): $\mathrm{GRM}=\frac{\tilde{X}\tilde{X}\prime }{ncol(\tilde{X})}$. Here, GRM is a matrix describing genomic relationships between genotypes, $\tilde{X}=\left\{\left[X_{im}-mean\left(X_{im}\right)]/sd(X_{im}\right)\right\}$ is a matrix of centered and scaled SNP-based genotypes ($X_{im}\in \left\{0,1,2,3,4\right\}$ counts the number of copies of the reference allele at the *m^th^* loci. Subtracting the $mean\left(X_{im}\right),$ centers the SNP-based genotypes to a null mean and dividing by the SNP standard deviation, $sd(X_{im})$, scale SNP-based genotypes to unit variance). Finally, division by the number of SNP-based genotypes, $ncol(\tilde{X})$, makes the average diagonal value of GRM equal to one. We use this matrix to quantify genomic relationships and to derive principal components, the later were computed by applying the *eigen()* R-function to *GRM*.

### Statistical analyses

We use whole-genome regression models (Meuwissen *et al.* 2001; de los Campos *et al.* 2013) for estimation of marker effects and variance component analyses and for assessment of prediction accuracy. The general form of the statistical model used was as follows:

$$y_{ijk}=\mu +\overset{5}{\underset{h=1}{\sum }}PC_{hi}\gamma_{h}+b_{j}+g_{i}+ge_{ij}+\varepsilon_{ijk}$$ [1]

where $y_{ijk}$ is a phenotypic score (for either late blight or common scab) of the *k^th^* replicate of the *i^th^* genotype collected in year *j*, μ is an intercept, $\overset{5}{\underset{h=1}{\sum }}PC_{hi}\gamma_{h}$ is a regression on the first five SNP-derived principal components, $b_{j}$ are year effects, $g_{i}$ is the main effect of the *i^th^* genotype (alternative specifications of this effect are discussed below), $ge_{ij}$ represents a genotype-by-year interaction and $\varepsilon_{ijk}$ are error terms, which were treated as normal, independently and identically distributed (*iid*) with year-specific variances, that is $\varepsilon_{ijk}\begin{array}{c} iid\\ ∼ \end{array}N\left(0,\sigma_{j}^{2}\right)$.

Year had seven levels for late blight and nine levels for common scab (2009, 2010,.., 2017) and was treated as a random effect. Genetic and genetic-by-year interactions were also modeled as random effects. We considered four specifications for modeling the main effect of genotypes:

- **Genotype effect.** In this specification we assumed that the main effects of the genotypes where *iid* draws from normal distributions $g_{i}\begin{array}{c} iid\\ ∼ \end{array}N\left(0,\sigma_{g}^{2}\right)$. In this specification, no genetic information (SNPs) was used and no assumptions about gene action (additive, dominance, epistasis) were made. This specification was used as a baseline for a model that could be fitted without having genomic information. The other three specifications included genotypes as inputs.
- **Additive model (A)**.  Here, the main effect of the genotype was represented using a linear combination of the marker genotypes, that is $g_{i}=\overset{4110}{\underset{m=1}{\sum }}{\tilde{X}}_{im}\alpha_{m}$ where ${\tilde{X}}_{im}=[X_{im}-mean\left(X_{im}\right)]/sd(X_{im})$ were centered and scaled genotypes code at the *m^th^* SNP in the *i^th^* genotype and $\alpha_{m}$ is the additive effect of the markers.
- **Additive + Dominance (A+D)**. In this case, the main effects of genotypes have an additive component plus one that accounted for dominance; therefore in this model $g_{i}=\overset{4110}{\underset{m=1}{\sum }}{\tilde{X}}_{im}\alpha_{m}+\overset{4110}{\underset{m=1}{\sum }}{\tilde{D}}_{im}d_{m}$ where ${\tilde{D}}_{im}=\left[D_{im}-mean\left(D_{im}\right)\right]/sd\left(D_{im}\right)$ are (centered and standardized) dummy variables for heterozygous loci, here $D_{im}$=1 (=0) indicates that the *m^th^* SNP of the *i^th^* genotype was in heterozygous (homozygous) state and $d_{m}$ is the dominant effect of the markers.
- **General model (G)**. Here, $g_{i}=\overset{4110}{\underset{m=1}{\sum }}\overset{4}{\underset{n=0}{\sum }}{\tilde{W}}_{imn}\gamma_{mn}$, where ${\tilde{W}}_{imn}$ are (centered and standardized) dummy variables for genotypes carrying *n* copies of the reference allele and $\gamma_{mn}$ is the general effect of the markers. Since there are up to five distinct genotypes (0,1,…,4) this model includes up to four degree of freedom per locus. This parameterization allows for any form of interactions of alleles within locus; thus, it can be considered the most general specification for a model accounting for additive and dominance effects.

### Prior distributions for effects

***Marker effects*** (including both additive, dominance and those of the G model) were treated as random. We considered two prior distributions of effects: (i) treating SNP effects as draws from normal distributions with null mean and model-specific variances (*i.e.*, there were separate variances for additive and dominance), this approach was implemented using the Bayesian Ridge Regression (BRR) specification in the Bayesian Generalized Linear Regression (BGLR) R-package (Pérez and de los Campos 2014), and (ii) a Bayesian shrinkage-variable selection method (BayesB, Meuwissen *et al.* 2001). As with BRR, in BayesB different regularization parameters (probabilities of non-null effects and scale parameters) were assigned to effects in additive and dominance. BayesB was implemented in BGLR using the “BayesB” keyword for the model argument of the linear predictor.

***Genotype-by-year effects*** ($ge_{ij}$) were treated as IID normal with mean zero and variance common to all the interactions, that is, $ge_{ij}\begin{array}{c} iid\\ ∼ \end{array}N\left(0,\sigma_{ge}^{2}\right).$

#### Sequence of models:

Using the specifications described above, we produced a sequence of models designed to quantify the amount of variance explained (and the contribution to prediction accuracy) of each of the terms entering in the model of expression [1]. The sequence of models considered is summarized in Table 1.

**Table 1:** Sequence of models

Model # (label)*^a^*	Effects Included							
	Year	Genotype^*b*^	PC*^c^*	Additive*^d^*	Dominance^*e*^	General^*f*^	Genotype-by -Year^*g*^	Error
**M1**	×							×
**M2**	×	×						×
**M3**	×	×					×	×
**M4**	×	×	×				×	×
**M5 (A)**	×		×	×			×	×
**M6 (A+D)**	×		×	×	×		×	×
**M7 (G)**	×		×			×	×	×

a M1-M7 are model numbers. ^*b*^Random effect of the genotype (no SNPs used, no assumption about gene action are made).

c Principal components, ^*d*^linear regression on allele content (0/1/2/3/4), ^*e*^Simple dominance (1 degree of freedom per locus representing heterozygous) and ^*f*^General model for additive + dominance (with up to 4 degrees of freedom per locus). ^*g*^Genotype-by-year interaction. An ‘×’ indicates that the effects was included in the model.

We used the whole-genome regression models described above for three purposes: (1) estimation of variance components, (2) identification of variants with high contribution to additive variance and (3) assessment of prediction accuracy in cross-validation.

#### Variance components:

The amount of variance accounted for by each of the terms included in the model was estimated using the methods described in de los Campos *et al.* (2015) and Lehermeier *et al.* (2017) (see Supplementary File S1 for further details). We use these methods to decompose the total phenotypic variance into components due to year, genetics factors, genotype-by-year interactions (G×E) and within-year error variance. We also use this approach to assess the relative contribution of SNP-additive and dominance effects.

#### Identification of SNPs With a sizable contribution to additive variance:

Response to selection is directly proportional to additive variance (Falconer and Mackay 1996). Thus, in GS, the single-loci additive variance represents a natural metric to assess the relative importance of individual loci from a breeding perspective. Under linkage equilibrium, the contribution of each locus to additive variance is given by $Var\left(X_{im}\alpha_{m}\right)=Var\left(X_{im}\right)\alpha_{m}^{2}$. In our case, genotypes were standardized to unit variance; therefore, $Var\left(X_{im}\alpha_{m}\right)=\alpha_{m}^{2}$. We used samples from the posterior distribution of SNP effects from the A model to assess the contribution of individual loci to additive variance. (Further details about how these quantities were computed can be found in the script provided with the Supplementary File S1). It is important to note that this measure does not account for linkage disequilibrium; thus it can only be taken as a proxy of the contribution of a locus to additive variance.

#### Prediction accuracy evaluation:

We implemented two cross-validation schemes. First, we used a fivefold cross-validation, assigning genotypes to folds. When using this approach all the phenotypic records of a genotype are assigned to either training or testing populations. Thus, this approach yields an estimate of the prediction accuracy that can be achieved predicting the performance of genotypes that have not been evaluated in field trials (*i.e.*, prediction based on genotype data only) and is equivalent to the method labeled as Cross-Validation one (CV1) in Burgueño *et al.* (2012). For this scheme, genotypes were assigned to folds completely at random and the fivefold Cross Validation (CV) was repeated 100 times to obtain accurate estimates of the average prediction correlation and its standard deviation.

In a second prediction scheme (CV2), we assigned years to folds (*i.e.*, there as many folds as years). Thus, when analyzing the *j^th^* fold, data from the *j^th^* year was assigned to testing and data from all the other years was used for training. This CV approach yields an estimate of the prediction accuracy that can be achieved when attempting to predict future year performance based on past data. Note that in this case, unlike CV1, when predicting data for the *i^th^* genotype on the *j^th^* year all the data from the *i^th^* genotype collected in other years was part of the training dataset.

In both CV schemes prediction accuracy was evaluated by computing the within-year correlation between phenotypes and CV predictions.

#### Software:

All the analyses were conducted using R (The R Development Core Team 2010). Models were fit using the BGLR-R package. For each model, we ran the Gibbs sampler algorithm for a total of 33,000 cycles, discarding the first 3,000 samples for burn-in; one of every five of the remaining samples was saved and used to estimate posterior means and standard deviations.

### Data availability

Scripts demonstrating how each of the models were implemented in BGLR are given in File S1. The genotype and phenotype data are provided in Files S2 and S3 for late blight and common scab, respectively. File S4 contains Tables S1-S5. Supplemental material is available at Figshare: https://doi.org/10.6084/m9.figshare.6336911 and https://doi.org/10.25387/g3.6262214.

## Results

**Figure 1:**
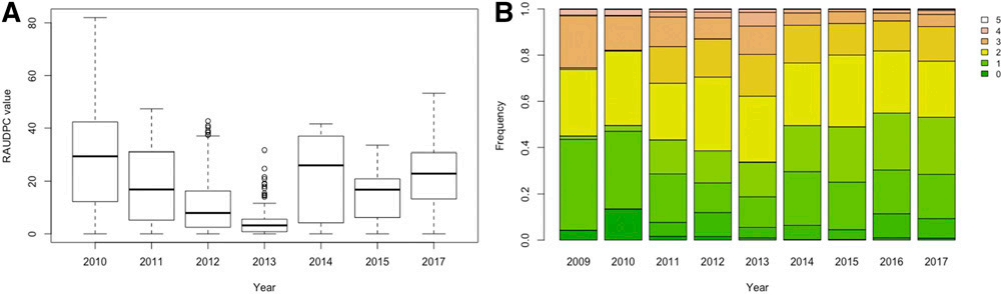
Boxplot of late blight scores (A, relative area under the disease progress curve- RAUDPC) and bar plot for common scab scores (B, 0-5 rating scale).

The distribution of late blight and common scab infection varied substantially between years (Figure 1). In general, RAUDPC median values decreased from 2010 to 2012, with US-22 as the prevalent late blight strain on infected plants. In subsequent years, a differential response for late blight resistance was observed when US-23 was the prevalent strain. Disease pressure changes, together with the environment fluctuations between years contribute to explain the phenotypic variation observed for the late blight resistance response. Similarly, for common scab, a reduced frequency of resistant genotypes (0-1 score) was observed from 2009 until 2013, having at the same time an increasing number of intermediate susceptible genotypes (2-3 scores). Since 2013 and until 2017, an increased frequency of common scab resistant genotypes was observed (Figure 1).

**Figure 2:**
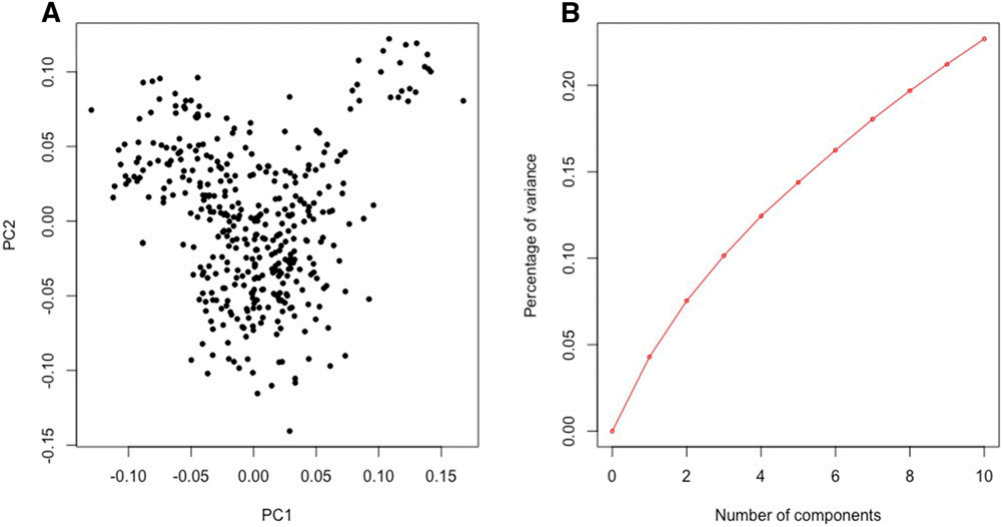
Principal component analysis of the Michigan State University’s potato breeding genotypes derived from 4,110 SNPs: loadings on the first two marker-derived principal components (A) and proportion of variance explained by the top 10 principal components (B).

**Figure 3:**
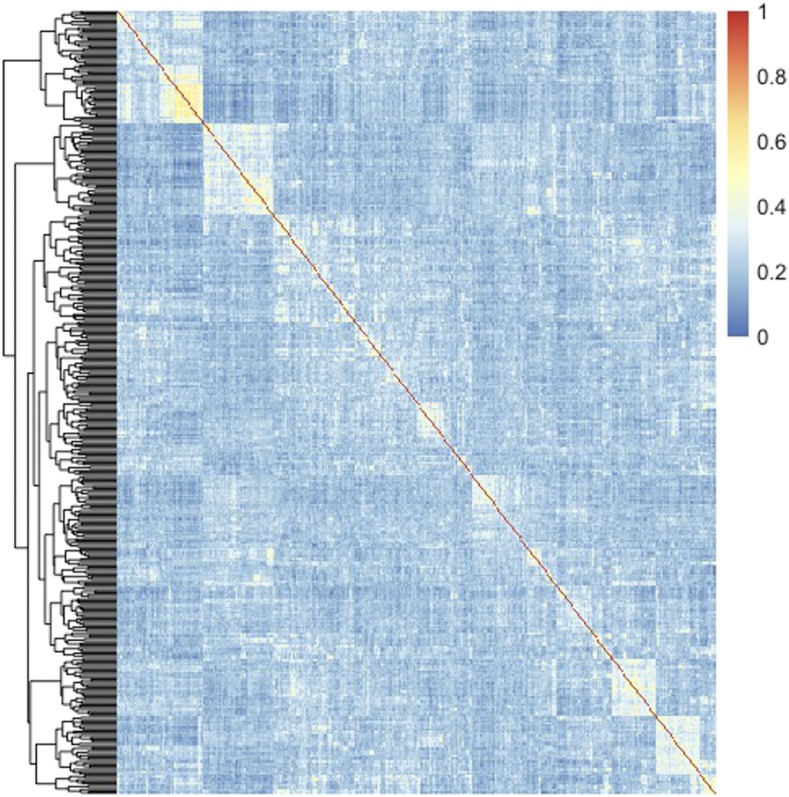
Heatmap of the genomic relationship matrix (GRM) from the Michigan State University’s potato breeding genotypes.

A principal component (PC) analysis showed that potato genotypes clustered in two groups, one involving 391 genotypes, and a small one including 22 genotypes (Figure 2). The eigenvalues associated to the first two PCs explained about 8% of the total genotypes variance (Figure 2). A cluster analysis using a correlation matrix derived from SNP markers supports the PC-analysis results (Figure 3). The heatmap also reveals that the strength of genomic relationships among the different materials is relatively small (the clear majority of the genotypes have genomic relationships with other genotypes smaller than 0.1, with only a few genotypes showing relationships comparable to parent-offspring or full-sib relations, *i.e.*, 0.5, Figure 3).

### Variance Components Estimates

The variance components analyses for late blight resistance (Table 2 and Table S2 in File S4) revealed that year explained roughly 25% of the variance in disease scores. For this trait, and taking as a reference the model M3, the main effect of genotype explained about 34% of the variance, genotype-by-year interactions explained 25% of the variance and the error term explained roughly 14% of the variance in late blight scores. These results suggest that a substantial proportion of within-year variance in late blight scores (roughly 70%, computed as 0.34/[0.34+0.144]) can be explained by main effects of genotypes. For *late bligh*t, the amount of genetic variance captured by the A model was roughly 94% of the variance captured by the G model (computed as 0.330/0.352).

**Table 2:** Variance components estimates (posterior standard deviation) derived from BayesB model for late blight and common scab resistance by model. Phenotypic scores were standardized to unit variance; hence estimates can be interpreted as the proportion of variance explained by each component. Results obtained with the fully Gaussian model (BRR) are presented in Table S2

Model # (label)*^a^*	*Year*	*Genetic*						Genotype-by-year*^g^*	Error
		*Genotype*	*Marker-derived*				Total genetic*^*f*^*		
			*PC**^b^*	Additive^*c*^	Dominance^*d*^	General^*e*^			
Late blight									
M1	0.266 (0.021)								0.735 (0.025)
M2	0.256 (0.014)	0.434 (0.018)					0.434 (0.018)		0.303 (0.011)
M3	0.250 (0.027)	0.340 (0.031)					0.340 (0.031)	0.253 (0.019)	0.144 (0.006)
M4	0.244 (0.026)	0.265 (0.028)	0.096 (0.024)				0.351 (0.032)	0.251 (0.018)	0.144 (0.006)
M5 (A)	0.240 (0.027)		0.135 (0.069)	0.292 (0.051)			0.330 (0.035)	0.275 (0.020)	0.144 (0.006)
M6 (A+D)	0.240 (0.027)		0.135 (0.062)	0.166 (0.063)	0.141 (0.049)		0.340 (0.034)	0.267 (0.020)	0.144 (0.006)
M7 (G)	0.249 (0.028)		0.107 (0.043)			0.280 (0.034)	0.352 (0.033)	0.251 (0.019)	0.144 (0.006)
Common Scab									
M1	0.033 (0.006)								0.971 (0.022)
M2	0.030 (0.004)	0.456 (0.016)					0.456 (0.016)		0.523 (0.012)
M3	0.029 (0.005)	0.440 (0.021)					0.440 (0.021)	0.059 (0.009)	0.483 (0.013)
M4	0.029 (0.006)	0.419 (0.021)	0.030 (0.012)				0.447 (0.023)	0.056 (0.009)	0.485 (0.013)
M5 (A)	0.031 (0.006)		0.132 (0.087)	0.507 (0.076)			0.443 (0.025)	0.061 (0.009)	0.485 (0.013)
M6 (A+D)	0.031 (0.006)		0.107 (0.073)	0.356 (0.087)	0.151 (0.060)		0.448 (0.024)	0.057 (0.009)	0.485 (0.013)
M7 (G)	0.031 (0.006)		0.051 (0.030)			0.442 (0.031)	0.451 (0.023)	0.056 (0.009)	0.484 (0.013)

a M1-M7 are model numbers (label). The effects included in each of them are described in the columns.

b Principal components, ^*c*^linear regression on allele content (0/1/2/3/4), ^*d*^Simple dominance (1 degree of freedom per locus representing heterozygous) and ^*e*^General model for additive + dominance (with up to 4 degrees of freedom per locus). ^*f*^Total genetic variance, ^*g*^Genotype-by-year interaction.

For common scab (Table 2 and Table S2 in File S4) the main effect of genotype explained about 44% of the total variance, year and genotype-by-year effects explained only 3% and 6% of the total variance, respectively, and the error term accounted for almost one half (48%) of the variance in disease scores. For common scab we also observed that the amount of genetic variance captured by the A model was very similar to the one captured with the G model.

The proportion of the total genetic variance that could be attributed to the first-5 PCs was substantial for late blight (∼30%, computed as 0.107/0.352) and low for common scab (∼10%, 0.051/0.451).

**Figure 4:**
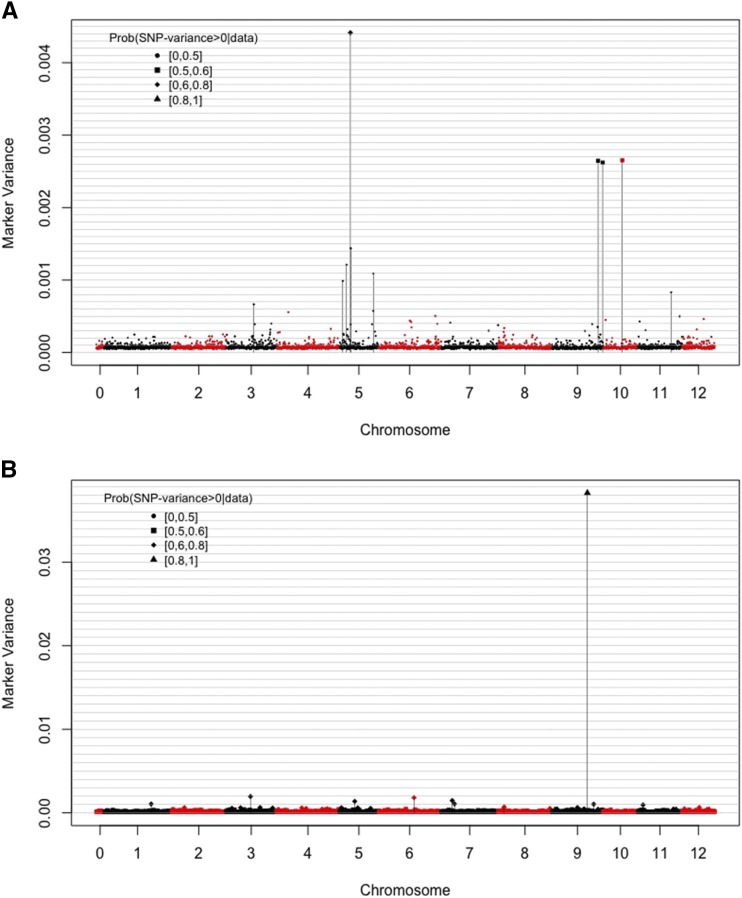
Estimated SNP-variances derived from BayesB model using the additive model for late blight (A) and common scab (B). (In both cases, phenotypes were disease scores standardized to a variance equal to one. Vertical lines indicate the positions of the top-10, according to estimated SNP-variance markers).

For the A model (fitted using BayesB), we computed single-locus variances and used these estimates as proxies for the SNP relevance (Figures 4a and 4b). Additionally, we report in Figures S1 and S2, linkage disequilibrium (LD) plots for the 10 leading SNPs (*i.e.*, those with the larger single-SNP variance) for each trait. For both pathogens, there were a few regions with large single-SNP-variance. Specifically, for late blight, there were multiple SNPs distributed across the potato chromosomes (Figure 4a and Table S3 in File S4) with a sizable contribution to variance, suggesting that multiple genes contribute to the resistant phenotype. Conversely, for common scab, there was one SNP, located in chromosome IX (Figure 4b and Table S4 in File S4), that stands out for its contribution to variance and a few SNPs with a moderate contribution to phenotypic variance.

The results from the first cross-validation analysis (CV1) yielded an estimated prediction correlation of about 0.31 for late blight resistance using the G model. For this trait, there was a relatively small, albeit significant, increase in prediction correlation for the G model relative to the A model. Likewise, there was a slight superiority of BayesB over BRR (Table 3). In the case of common scab, the A model (with a prediction correlation of ∼0.27) outperformed the A+D (correlation ∼0.26) and G (correlation ∼0.22) models. These results agree with the variance component analyses results, where we also found evidence of a slightly higher relevance of non-additive effects in the case of late blight, compared to common scab.

**Table 3:** Cross-validation correlations obtained with BRR and BayesB models by trait and model

				Proportion of times that the model in row gave a higher correlation than the model in columns					
		CV-Correlation		BRR*^a^*			BayesB*^a^*		
Prior*^a^*	Model # (label)*^b^*	Average*^c^*	SD*^d^*	M5 (A)	M6 (A+D)	M7 (G)	M5 (A)	M6 (A+D)	M7 (G)
Late Blight									
BRR	M5 (A)	0.258	0.023		0.96	0.00	0.33	0.91	0.00
	M6 (A+D)	0.241	0.023	0.04		0.00	0.04	0.57	0.00
	M7 (G)	0.312	0.017	1.00	1.00		1.00	1.00	0.5
BayesB	M5 (A)	0.260	0.024	0.67	0.96	0.00		0.94	0.00
	M6 (A+D)	0.240	0.024	0.09	0.43	0.00	0.06		0.00
	M7 (G)	0.313	0.017	1.00	1.00	0.50	1.00	1.00	
Common Scab									
BRR	M5 (A)	0.268	0.025		0.81	0.99	0.1	0.55	1.00
	M6 (A+D)	0.259	0.023	0.19		0.99	0.07	0.20	0.99
	M7 (G)	0.218	0.022	0.01	0.01		0.00	0.02	0.63
BayesB	M5 (A)	0.278	0.026	0.9	0.93	1.00		0.91	1.00
	M6 (A+D)	0.265	0.025	0.45	0.8	0.98	0.09		0.98
	M7 (G)	0.216	0.022	0	0.01	0.37	0.00	0.02	

a BRR uses a Gaussian prior for effects, BayesB uses a prior that has a point of mass at zero and a scaled-t slab.

b A: Additive model, A+D: additive+dominance; G: general model (with up to 4 degrees of freedom per locus).

c Average from 100 cross-validations.

d Standard deviation.

Note that in Table 3 we only included results from models using genotypes. Results from other models (*e.g.*, M2 and M3) are not presented because in CV1 they render zero within-year correlation. This happens because in CV1 predictions are entirely depending on borrowing of information between genotypes, a feature that is not possible in models that do not use genotype or pedigree information.

The results from the second cross-validation (*i.e.*, where years were assigned to folds, CV2) yielded higher estimates of prediction accuracy than those obtained in CV1 (Table 4 and Table S5 in File S4). This happens because in CV2 there is within-genotype borrowing of information across years. For late blight, prediction correlations ranged from 0.41 to 0.74, depending on the model and year. Likewise, for common scab, we obtained correlations ranging from 0.46 to 0.76. For both traits, the across-year average correlations showed small differences between models (with a slight superiority in favor of the G model).

**Table 4:** Year cross-validation correlations obtained with BayesB model by trait and model

Year	Model # (label)*^a^*				
	M2	M3	M5 (A)	M6 (A+D)	M7 (G)
**Late blight**					
2010	0.551	0.537	0.463	0.465	0.517
2011	0.652	0.658	0.608	0.611	0.642
2012	0.583	0.604	0.586	0.586	0.624
2013	0.422	0.415	0.484	0.485	0.492
2014	0.621	0.596	0.633	0.640	0.655
2015	0.719	0.730	0.678	0.696	0.745
2017	0.508	0.504	0.471	0.491	0.506
**Average**	0.579	0.578	0.560	0.568	0.597
**SD**	0.098	0.104	0.087	0.089	0.095
**Common scab**					
2009	0.459	0.460	0.472	0.471	0.466
2010	0.520	0.522	0.535	0.533	0.517
2011	0.610	0.611	0.622	0.625	0.618
2012	0.625	0.628	0.628	0.626	0.634
2013	0.750	0.759	0.731	0.737	0.750
2014	0.615	0.611	0.634	0.636	0.635
2015	0.649	0.653	0.666	0.671	0.659
2016	0.639	0.639	0.652	0.647	0.647
2017	0.508	0.510	0.519	0.520	0.515
**Average**	0.597	0.599	0.606	0.607	0.605
**SD**	0.088	0.090	0.082	0.083	0.089

a M2 includes year and genotype (no SNP information); M3: extends M2 with the addition of genotype-by-year interaction; M5 includes year, first 5 marker-derived PCs, additive effect of SNPs and genotype-by-year interaction; M6 expands M5 by adding the effects of dominance; M7 includes year, 5-PCs, genotype-by-year interactions and SNPs with up to 4 degrees of freedom per locus (‘General’ model).

## Discussion

Genomic selection has been quickly adopted for breeding in diploid species (Heffner *et al.* 2009; Daetwyler *et al.* 2013; de los Campos *et al.* 2013). However, the volume of research and the adoption of the GS technology for breeding of polypoid species has been much more limited (*e.g.*, Habyarimana *et al.* 2017; Sverrisdóttir *et al.* 2017). In this study, we demonstrate how genomic models commonly used in GS of diploid organisms can be applied for the analysis and prediction of disease susceptibility in autotetraploid potato.

Our results indicate that a sizable fraction of the within-year inter-individual differences in disease resistance (about 0.46 ± 0.04 for late blight and 0.45 ± 0.02 for common scab) can be explained using 4,110 codominant SNPs from the Infinium 8303 Potato Array used in this study. These moderately high genomic heritability estimates for complex disease phenotypes indicates that, in principle, genomic prediction could be used successfully to select for resistance to late blight and common scab.

Previous studies have reported heritability estimates for these traits; however, differences in the nature of the genetic materials (diploid *vs.* tetraploid, hybrids *vs.* genotypes) and of the environmental conditions (natural *vs.* induced infection) makes the comparisons across studies difficult (Nelson 1978; Braun *et al.* 2017b). For instance, Haynes and Christ (1999) reported much higher heritability estimates for late blight resistance (0.8), but this study was based on diploid hybrids. For the same trait, estimates of heritability obtained using tetraploid genotypes are closer to the ones reported here (ranging from 0.31 to 0.69, Pajerowska-Mukhtar *et al.* 2009; Solano *et al.* 2014).

For common scab, previous heritability estimates are also highly variable, depending on the genetic material and the environmental conditions. For instance, using diploid potatoes derived from a cross between wild relatives (*S. phureja* × *S. stenotonum*) and cultivated potatoes (di-haploid *S. tuberosum* × *S. chacoense*), Haynes *et al.* (2009) and Braun *et al.* (2017b) reported broad sense heritability estimates ranging from 0.18 to 0.72 for different environments. However, studies involving tetraploid potatoes have reported higher heritability estimates with values ranging from 0.32 to 0.93 (Haynes *et al.* 1997; Bradshaw *et al.* 2008; Tai *et al.* 2009). More recently, 18 dedicated common scab and standard breeding program trials were conducted in fields with high disease pressure. The broad sense heritability estimates reported from these studies ranged from 0.75 to 0.90 for dedicated common scab trials and from 0.06 to 0.82 for standard breeding programs trials involving advanced breeding materials (Navarro *et al.* 2015).

The amount of variance in disease resistance that could be attributed to genotype-by-year interactions was high for late blight and very small for common scab. These differences are likely to be due to the different nature and characteristics of infection on the fields used to evaluate late blight and common scab. Specifically, for late blight, the mean scores varied substantially between years (*e.g.*, it was clearly low in 2013) reflecting changes on the late blight aggressiveness and late blight genotypes present in different years, resulting in a large extent of genotype-by-year interactions for this pathogen. On the other hand, our common scab data were generated in a nursery that has been used to evaluate common scab resistance in potato breeding genotypes for several years. Consequently, there was less variability in the mean scores across years and therefore we observed substantially less extent of G×E. A similar result was reported under comparable conditions by Murphy *et al.* (1995). Results based on fields trials performed in different locations for this pathogen have shown much higher variability over the years (Haynes *et al.* 2009).

The comparison of the genomic variance estimates obtained with the A model and those obtained with the G model suggest that, for both pathogens, a sizable fraction of the total genetic variance (0.94 and 0.98, for late blight and common scab, respectively) can be captured by an additive model (Table 2 and Table S2 in File S4). The amount of genetic variance captured by the A model reflects an estimate of the variance that can be captured by regression on allele content (*i.e.*, by allele substitution effects). However, when dominance is included in the model (A+D), the estimated ‘additive variance’ no longer represents the variance explained by allele substitution effects; therefore, the additive component in the A+D model is smaller than the additive component estimated with the A model.

While our variance component estimates indicate that most of the genetic variance can be captured by an A model, our cross-validation analysis suggests that accounting for non-additive effects could improve prediction accuracy by a small but statistically significant margin in the case of late blight. These results agree with the theory that suggests that dominance and epistasis are expected to contribute to the expression of traits subjected to directional selection or those affecting the plant fitness such as late blight resistance (Killick and Malcolmson 1973). This may explain why the G model captured slightly more variance and predicted slightly more accurately late blight scores than the A model.

The presence of linkage disequilibrium (LD) between loci makes the partition of the total genetic variance into (orthogonal) locus-specific components not possible (de los Campos *et al.* 2015). However, it is worth looking at the relative size of estimated effects to explore features of the genetic architecture of the trait. We did this by inspecting the estimated SNPs variances (Figure 4). Overall, the proportion of variance explained by individual SNPs was low, reinforcing the idea that resistance to both common scab and late blight is polygenic. However, there were some SNPs with relatively large SNP-variances for both late blight (located mainly in chromosomes V and IX) and common scab (located in chromosome IX). (Figure 4, Table S3 and S4 in File S4). For late blight resistance, multiple quantitative trait loci (QTL) have been reported across the 12 potato chromosomes in tetraploid and diploid potato populations (Tiwari *et al.* 2013). Most of these major QTL are located in chromosomes III, IV, V, VII, XI and XII, characterized for harboring hotspot regions for resistance to late blight and other pathogens, not only for genes involved in quantitative resistance such as R genes, but also for genes involved in qualitative resistance (Malosetti *et al.* 2007; Pajerowska-Mukhtar *et al.* 2009; Álvarez *et al.* 2017). For instance, genes involved in carbohydrate metabolism such as sucrose synthase (Table S3 in File S4) play an active role in the defense response elicitation. Sucrose synthesis down-regulation has been described in the *Capsicum annuum - Phytophthora nicotianae* pathosystem, showing a decreasing concentration after challenging with beta-aminobutyric acid (BABA) and priming the synthesis of metabolites associated with the production of defense-related compounds (Stamler *et al.* 2015). Additionally, these results validate earlier QTL reports obtained from MSU-derived populations using potato varieties carrying different late blight resistance genes coming from species previously used in resistance breeding such as *S. demissum* and *S. berthaultii* (Massa *et al.* 2015; N. Manrique-Carpintero, personal communication).

For common scab resistance, our results suggest an additive resistance effect with a clear major-effect SNP located on chromosome IX. This SNP is associated with a WRKY transcription factor known for their role in the modulation of the resistance responses in systemic and acquired plant resistance, activating or repressing the transcription of genes involved in the synthesis of defense related-proteins such as R proteins (Pandey and Somssich 2009; Buscaill and Rivas 2014). In addition to the loci discussed above, we were also able to identify additional SNPs with a sizable contribution to variance across the potato chromosomes (Figure 4). Interestingly, the SNP in the WRKY gene that appeared to have a sizable contribution to inter-individual differences in common scab resistance is located in a region where LD is relatively weak (see Figure S2).

For instance, we found one SNP located in chromosome III (Table S4 in File S4) associated with the primary metabolism-related protein fructokinase, whose concentration increases under pathogen attack as a mechanism to reduce the costs attributed to the defense response in soil-borne pathogens (Zimaro *et al.* 2011). Likewise, in chromosome V, we found one SNP related to the RNA synthesis-related protein DEAD-box ATP-dependent RNA helicase, reported for its role in plant resistance by enhancing the defense response in both necrotrophic and biotrophic pathogens (Li *et al.* 2008). Overall, the evidence we found support the hypothesis that resistance to common scab involves multiples mechanisms of defense including the activation of genes related to systemic and R gene-mediated resistance.

There are few studies reporting QTL for common scab resistance. For instance, two QTL located in chromosome XI were detected in a diploid parental-derived population for the percentage of surface area infected and lesion type caused by common scab, explaining 21% and 18.2% of the total phenotypic variance, respectively (Braun *et al.* 2017a). For tetraploid populations, Amplified Fragment Length Polymorphisms (AFLPs) and Simple Sequence repeats (SSRs) markers have been used to establish an association between potato genotypes and the common scab resistance phenotype in a tetraploid bi-parental derived-population. Two copies of a dominant allele were detected in a QTL localized in chromosome II, explaining 8.1 and 7.1% of the phenotypic variance, respectively. A second QTL was localized in chromosome VI explaining 6.9% of the total phenotypic variance (Bradshaw *et al.* 2008). Therefore, the large-variance SNP detected in this study represents a new genomic region associated with common scab resistance, providing a framework for the development of molecular markers for marker-assisted selection and understand the genetics behind common scab resistance.

Our variance component estimates suggest that for both, late blight and common scab, a sizable amount of inter-individual differences in disease resistance can be captured using whole-genome regressions. However, the successful implementation of GS requires being able to predict future outcomes from past data. We assessed this using two CV analyses. Our results are based on genotypes derived from potato breeding programs. Some of these genotypes are related through pedigrees and there is some level of population stratification. Therefore, the prediction accuracies reported in our study should be considered representative of the prediction accuracy that one may be able to achieve when applying GS to breeding populations.

We considered two different prediction problems and implemented different CV schemes to represent each prediction problem. Our first CV focused on the prediction of future scores from genotypes that were not evaluated in field trials (*i.e.*, prediction based on information from other genotypes). These analyses rendered moderately low CV-correlations (∼0.22-0.31 with some small differences between traits and models).

It is important to highlight that in CV1 the correlations reflect the prediction accuracy that can be achieved when predicting future phenotypes for genotypes that have not been evaluated in field trials. These predictions, although imperfect, could enable several rounds of rapid selection based on genotype data alone. The predictive correlation obtained in CV1 was about half of the correlation between phenotypes across years (compare results in Table 3 with those for M2 in Table 4). Thus, we conclude that with the array and sample size used in this study, the predictive accuracy for late blight and common scab scores obtained from a newly developed genotype that has been genotyped but not tested in the field is about half of the predictive power of a single phenotype record. If more than two selection cycles can be carried out per year, the reduction on generation interval that can be achieved with genomic prediction would overcome the lower accuracy and, eventually lead to faster yearly genetic gains.

Our second CV used years as folds; therefore, in this case, disease scores predictions for one-year data were obtained from the same genotypes over years. The results of the model based on year and genotype (M2), give a baseline estimates of the prediction accuracy that can be achieved with phenotypic prediction. In CV2, we obtained higher prediction correlations (0.56-0.61, Table 4) than with CV1. However, the performance of the genomic models was only slightly superior to predictions based on past phenotypes-only (*i.e.*, those that could be obtained with the M2 model). This result agrees with previous studies (*e.g.*, Crossa *et al.* 2010) that show that the benefits of genomic prediction are more important when predicting phenotypes of materials that have no (or very limited) data from previous field trials.

### Conclusions

We confirmed that a sizable fraction of inter-individual differences in late blight and common scab scores can be attributed to genetic factors and can be captured using whole-genome regressions. We found large genotype-by-year interactions for late blight and limited genotype-by-year interactions for common scab. For both late blight and common scab, we found that an additive model could account for a sizable (>90%) of the total genetic variance. However, for late blight, we found small (but statistically significant) gains in prediction accuracy when accounting for dominance. Our analyses confirm strong associations with disease resistance to SNPs in previously reported resistance hotspot regions for late blight and reported a novel locus that has a sizable contribution to common scab resistance. We demonstrated that prediction of disease resistance, using genomic prediction applied to autotetraploid potato, is feasible and can be implemented for SNP-based selection in potato breeding. Further research is needed to explore ways (larger sample size, more controlled environments, higher marker density) in which genomic prediction accuracy can be further improved.

## References

[bib1] Ahn Y. K. Park T.-H., 2013 Resistance to common scab developed by somatic hybrids between *Solanum brevidens* and *Solanum tuberosum.* Acta Agriculturae Scandinavica, Section B - Soil &. Plant Sci. 63: 595–603.

[bib2] Álvarez M. F. Angarita M. Delgado M. C. García C. Jiménez-Gomez J., 2017 Identification of Novel Associations of Candidate Genes with Resistance to Late Blight in *Solanum tuberosum* Group Phureja. Front. Plant Sci. 8: 1040 10.3389/fpls.2017.01040 28674545 PMC5475386

[bib3] Barone A., 2004 Molecular Marker-assisted Selection for Potato Breeding. Am. J. Potato Res. 81: 111–117. 10.1007/BF02853608

[bib4] Bradshaw J. E. Hackett C. A. Pande B. Waugh R. Bryan G. J., 2008 QTL mapping of yield, agronomic and quality traits in tetraploid potato (*Solanum tuberosum* subsp. tuberosum). Theor. Appl. Genet. 116: 193–211. 10.1007/s00122-007-0659-1 17938877

[bib5] Braun S. R. Endelman J. B. Haynes K. G. Jansky S. H., 2017 a Quantitative trait loci for resistance to common scab and cold-induced sweetening in diploid potato. Plant Genome 10(3): 1–9. 10.3835/plantgenome2016.10.0110 29293805

[bib6] Braun S. Gevens A. Charkowski A. Allen C. Jansky S., 2017 b Potato Common Scab: a Review of the Causal Pathogens, Management Practices, Varietal Resistance Screening Methods, and Host Resistance. Am. J. Potato Res. 94: 283–296. 10.1007/s12230-017-9575-3

[bib7] Burgueño J. Campos G. D. L. Weigel K. Crossa J., 2012 Genomic Prediction of Breeding Values when Modeling Genotype × Environment Interaction using Pedigree and Dense Molecular Markers. Crop Sci. 52: 707–719. 10.2135/cropsci2011.06.0299

[bib8] Buscaill P. Rivas S., 2014 Transcriptional control of plant defence responses. Curr. Opin. Plant Biol. 20: 35–46. 10.1016/j.pbi.2014.04.004 24840291

[bib9] Crossa J. de los Campos G. Pérez P. Gianola D. Burgueño J., 2010 Prediction of Genetic Values of Quantitative Traits in Plant Breeding Using Pedigree and Molecular Markers. Genetics 186: 713 LP-724. 10.1534/genetics.110.118521 20813882 PMC2954475

[bib10] Daetwyler H. D. Calus M. P. L. Pong-Wong R. de los Campos G. Hickey J. M., 2013 Genomic Prediction in Animals and Plants: Simulation of Data, Validation, Reporting, and Benchmarking. Genetics 193: 347 LP-365. 10.1534/genetics.112.147983 PMC3567728 23222650

[bib11] Dees M. W. Wanner L. A., 2012 In Search of Better Management of Potato Common Scab. Potato Res. 55: 249–268. 10.1007/s11540-012-9206-9

[bib12] de los Campos G. Hickey J. M. Pong-Wong R. Daetwyler H. D. Calus M. P. L., 2013 Whole-Genome Regression and Prediction Methods Applied to Plant and Animal Breeding. Genetics 193: 327 LP-345. 10.1534/genetics.112.143313 22745228 PMC3567727

[bib13] de los Campos G. Sorensen D. Gianola D., 2015 Genomic Heritability: What Is It? PLoS Genet. 11: e1005048 10.1371/journal.pgen.1005048 25942577 PMC4420472

[bib14] Drewnowski A. Rehm C. D., 2013 Vegetable Cost Metrics Show That Potatoes and Beans Provide Most Nutrients Per Penny. PLoS One 8: e63277 10.1371/journal.pone.0063277 23691007 PMC3654977

[bib15] Driscoll J. Coombs J. Hammerschmidt R. Kirk W. Wanner L., 2009 Greenhouse and field nursery evaluation for potato common scab tolerance in a tetraploid population. Am. J. Potato Res. 86: 96–101. 10.1007/s12230-008-9065-8

[bib16] Falconer D. Mackay T., 1996 *Introduction to Quantitative Genetics (4e)*. Pearson, London.

[bib17] FAOSTAT, 2016 FAOSTAT Database Collections Food and Agriculture Organization of the United Nations, Rome.

[bib18] Fry W. E., 1978 Quantification of General Resistance of Potato Cultivars and Fungicide Effects for Integrated Control of Potato Late Blight. Phytopathology 68: 1650–1655. 10.1094/Phyto-68-1650

[bib19] Gebhardt C. Ballvora A. Walkemeier B. Oberhagemann P. Schüler K., 2004 Assessing genetic potential in germplasm collections of crop plants by marker-trait association: A case study for potatoes with quantitative variation of resistance to late blight and maturity type. Mol. Breed. 13: 93–102. 10.1023/B:MOLB.0000012878.89855.df

[bib20] Habyarimana E. Parisi B. Mandolino G., 2017 Genomic prediction for yields, processing and nutritional quality traits in cultivated potato (*Solanum tuberosum* L.). Plant Breed. 136: 245–252. 10.1111/pbr.12461

[bib21] Haverkort A. J. Struik P. C. Visser R. G. F. Jacobsen E., 2009 Applied Biotechnology to Combat Late Blight in Potato Caused by *Phytophthora* *Infestans*. Potato Res. 52: 249–264. 10.1007/s11540-009-9136-3

[bib22] Haynes K. G. Christ B. J., 1999 Heritability of resistance to foliar late blight in a diploid hybrid potato population of *Solanum phureja*×*Solanum stenotomum*. Plant Breed. 118: 431–434. 10.1046/j.1439-0523.1999.00394.x

[bib23] Haynes K. G. Christ B. J. Burkhart C. R. Vinyard B. T., 2009 Heritability of Resistance to Common Scab in Diploid Potatoes. Am. J. Potato Res. 86: 165–170. 10.1007/s12230-009-9068-0

[bib24] Haynes K. G. Goth R. W. Young R. J., 1997 Genotype × Environment Interactions for Resistance to Common Scab in Tetrapioid Potato. Crop Sci. 37: 1163–1167. 10.2135/cropsci1997.0011183X003700040023x

[bib25] Heffner E. L. Sorrells M. E. Jannink J.-L., 2009 Genomic Selection for Crop Improvement. Crop Sci. 49: 1–12. 10.2135/cropsci2008.08.0512

[bib26] Hirsch C. N. Hirsch C. D. Felcher K. Coombs J. Zarka D., 2013 Retrospective View of North American Potato (*Solanum tuberosum* L.) Breeding in the 20th and 21st Centuries. G3: Genes, Genomes. Genetics 3: 1003–1013. 10.1534/g3.113.005595 PMC3689798 23589519

[bib27] Killick R. J. Malcolmson J. F., 1973 Inheritance in potatoes of field resistance to late blight [*Phytophthora infestans* (Mont.) de Bary]. Physiol. Plant Pathol. 3: 121–131. 10.1016/0048-4059(73)90028-3

[bib28] Kolasa K., 1993 The potato and human nutrition. Am. Potato J. 70: 375–384. 10.1007/BF02849118

[bib29] Lehermeier C. de los Campos G. Wimmer V. Schön C.-C., 2017 Genomic variance estimates: With or without disequilibrium covariances? J. Anim. Breed. Genet. 134: 232–241. 10.1111/jbg.12268 28508483

[bib30] Li D. Liu H. Zhang H. Wang X. Song F., 2008 OsBIRH1, a DEAD-box RNA helicase with functions in modulating defence responses against pathogen infection and oxidative stress. J. Exp. Bot. 59: 2133–2146. 10.1093/jxb/ern072 18441339 PMC2413282

[bib31] Lozano R. Ponce O. Ramirez M. Mostajo N. Orjeda G., 2012 Genome-Wide Identification and Mapping of NBS-Encoding Resistance Genes in *Solanum tuberosum* Group Phureja. PLoS One 7: e34775 10.1371/journal.pone.0034775 22493716 PMC3321028

[bib32] Malosetti M. Van Der Linden C. G. Vosman B. Van Eeuwijk F. A., 2007 A mixed-model approach to association mapping using pedigree information with an illustration of resistance to *Phytophthora infestans* in potato. Genetics 175: 879–889. 10.1534/genetics.105.054932 17151263 PMC1800631

[bib33] Massa A. N. Manrique-Carpintero N. C. Coombs J. J. Zarka D. G. Boone A. E., 2015 Genetic Linkage Mapping of Economically Important Traits in Cultivated Tetraploid Potato (*Solanum tuberosum* L.). G3: Genes, Genomes. Genetics 5: 2357–2364. 10.1534/g3.115.019646 PMC4632055 26374597

[bib34] Meuwissen T. H. E. Hayes B. J. Goddard M. E., 2001 Prediction of total genetic value using genome-wide dense marker maps. Genetics 157: 1819–1829. 11290733 10.1093/genetics/157.4.1819 PMC1461589

[bib35] Mosquera T. Alvarez M. F. Jiménez-gómez J. M. Draffehn A. Hofmann A., 2016 Targeted and Untargeted Approaches Unravel Novel Candidate Genes and Diagnostic SNPs for Quantitative Resistance of the Potato (*Solanum tuberosum* L.) to Phytophthora infestans Causing the Late Blight Disease. PLoS One 11: e0156254. 27281327 10.1371/journal.pone.0156254 PMC4900573

[bib36] Muktar M. S. Lübeck J. Strahwald J. Gebhardt C., 2015 Selection and validation of potato candidate genes for maturity corrected resistance to *Phytophthora infestans* based on differential expression combined with SNP association and linkage mapping. Front. Genet. 6: 1–19. 10.3389/fgene.2015.00294 26442110 PMC4585299

[bib37] Murphy A. M. De Jong H. Tai G. C. C., 1995 Transmission of resistance to common scab from the diploid to the tetraploid level via 4x-2x crosses in potatoes. Euphytica 82: 227–233. 10.1007/BF00029565

[bib38] Navarro F. M. Rak K. T. Banks E. Bowen B. D. Higgins C., 2015 Strategies for Selecting Stable Common Scab Resistant Clones in a Potato Breeding Program. Am. J. Potato Res. 92: 326–338. 10.1007/s12230-015-9435-y

[bib39] Nelson R. R., 1978 Genetics of horizontal resistance to plant diseases. Annu. Rev. Phytopathol. 16: 359–378. 10.1146/annurev.py.16.090178.002043

[bib40] Nowicki M. Foolad M. R. Nowakowska M. Kozik E. U., 2011 Potato and Tomato Late Blight Caused by *Phytophthora infestans*: An Overview of Pathology and Resistance Breeding. Plant Dis. 96: 4–17. 10.1094/PDIS-05-11-0458 30731850

[bib41] Pajerowska-Mukhtar K. Stich B. Achenbach U. Ballvora A. Lübeck J., 2009 Single Nucleotide Polymorphisms in the Allene Oxide Synthase 2 Gene Are Associated With Field Resistance to Late Blight in Populations of Tetraploid Potato Cultivars. Genetics 181: 1115 LP-1127. 10.1534/genetics.108.094268 PMC2651047 19139145

[bib42] Pandey S. P. Somssich I. E., 2009 The Role of WRKY Transcription Factors in Plant Immunity. Plant Physiol. 150: 1648–1655. 10.1104/pp.109.138990 19420325 PMC2719123

[bib43] Pérez P. de los Campos G., 2014 Genome-Wide Regression & Prediction with the BGLR Statistical Package. Genetics 198: 483–495. 10.1534/genetics.114.164442 25009151 PMC4196607

[bib44] Pomerantz A. Cohen Y. Shufan E. Ben-Naim Y. Mordechai S., 2014 Characterization of *Phytophthora infestans* resistance to mefenoxam using FTIR spectroscopy. J. Photochem. Photobiol. B 141: 308–314. 10.1016/j.jphotobiol.2014.10.005 25463683

[bib45] Solano J. Acuña I. Esnault F. Brabant P., 2014 Resistance to *Phytophthora infestans* in *Solanum tuberosum* landraces in Southern Chile. Trop. Plant Pathol. 39: 307–315. 10.1590/S1982-56762014000400005

[bib46] Sparks A. H. Forbes G. A. Hijmans R. J. Garrett K. A., 2014 Climate change may have limited effect on global risk of potato late blight. Glob. Change Biol. 20: 3621–3631. 10.1111/gcb.12587 24687916

[bib47] Stamler R. A. Holguin O. Dungan B. Schaub T. Sanogo S., 2015 BABA and *Phytophthora nicotianae* Induce Resistance to *Phytophthora capsici* in Chile Pepper (*Capsicum annuum*). PLoS One 10: e0128327 10.1371/journal.pone.0128327 26020237 PMC4447391

[bib48] Sverrisdóttir E. Byrne S. Sundmark E. H. R. Johnsen H. Ø. Kirk H. G., 2017 Genomic prediction of starch content and chipping quality in tetraploid potato using genotyping-by-sequencing. Theor. Appl. Genet. 130: 2091–2108. 10.1007/s00122-017-2944-y 28707250 PMC5606954

[bib49] Tai G. C. C. Murphy A. M. Xiong X., 2009 Investigation of long-term field experiments on response of breeding lines to common scab in a potato breeding program. Euphytica 167: 69–76. 10.1007/s10681-008-9862-7

[bib50] The Potato Genome Sequencing Consortium, 2011 Genome sequence and analysis of the tuber crop potato. Nature 475: 189–195. 10.1038/nature10158 21743474

[bib51] The R Development Core Team, 2010 R : A Language and Environment for Statistical Computing. Vienna, Austria: R Foundation for Statistical Computing. Available at: http://www.R-project.org.

[bib52] Tiwari J. K. Siddappa S. Singh B. P. Kaushik S. K. Chakrabarti S. K., 2013 Molecular markers for late blight resistance breeding of potato: an update. Plant Breed. 132: 237–245. 10.1111/pbr.12053

[bib53] VanRaden P. M., 2008 Efficient Methods to Compute Genomic Predictions. J. Dairy Sci. 91: 4414–4423. 10.3168/jds.2007-0980 18946147

[bib54] Wanner L. A., 2006 A survey of genetic variation in Streptomyces isolates causing potato common scab in the United States. Phytopathology 96: 1363–1371. 10.1094/PHYTO-96-1363 18943669

[bib55] Wanner L. A. Kirk W. W., 2015 Streptomyces – from basic microbiology to role as a plant pathogen. Am. J. Potato Res. 92: 236–242. 10.1007/s12230-015-9449-5

[bib56] Zimaro T. Gottig N. Garavaglia B. S. Gehring C. Ottado J., 2011 Unraveling Plant Responses to Bacterial Pathogens through Proteomics. J. Biomed. Biotechnol. 2011: 354801 10.1155/2011/354801 22131803 PMC3216475

